# Supplementary material for: ZEB1 controls a lineage-specific transcriptional program essential for melanoma cell state transitions
Source: Oncogene. 2024 Mar 22;43(20):1489–505. doi: 10.1038/s41388-024-03010-7 (PMC11090790; doi:10.1038/s41388-024-03010-7)
Supplement: Supplementary file 1 — Supplementary figures and legends [file 41388_2024_3010_MOESM1_ESM.pdf]

Supplementary Figure 1

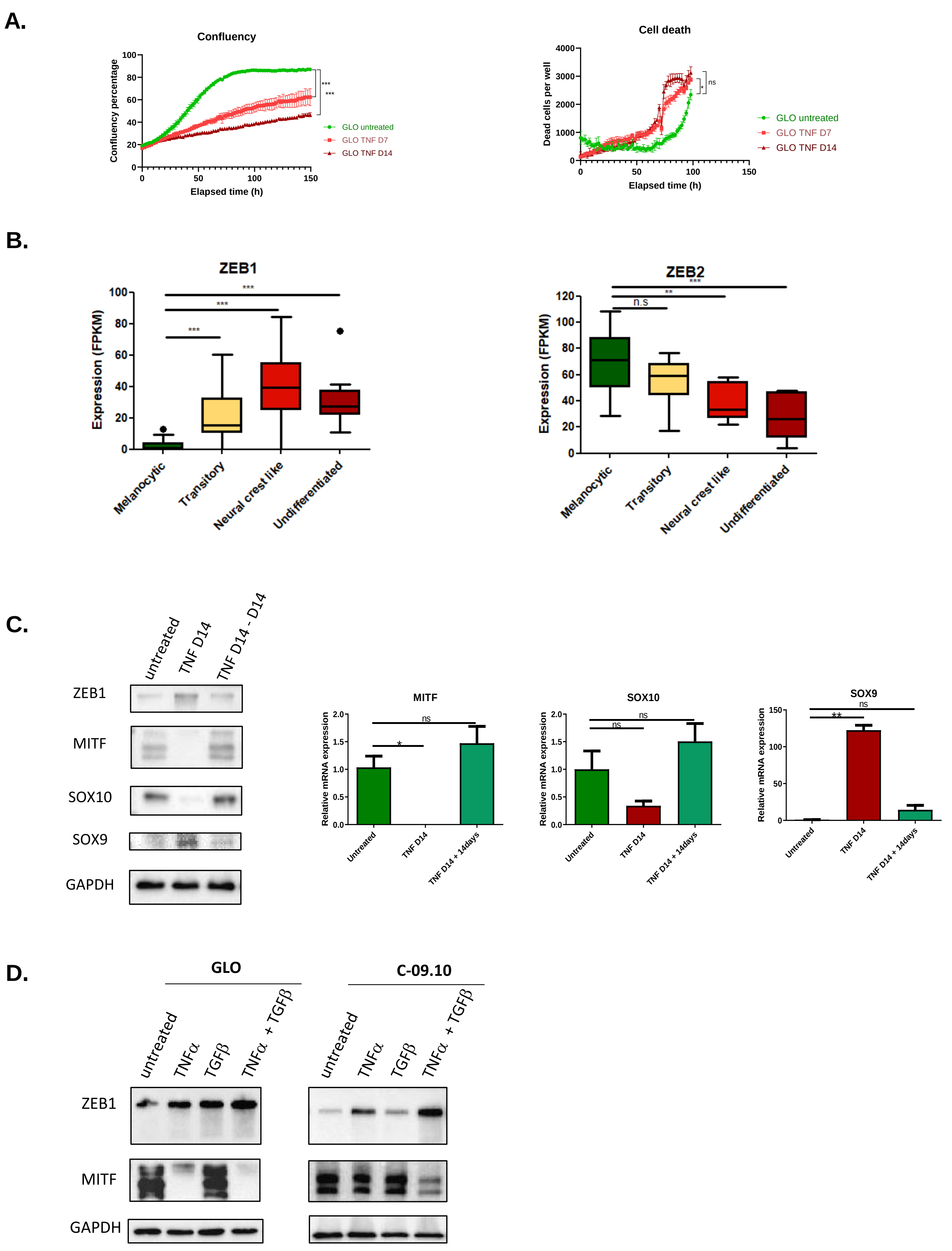

Supplementary Figure 1. TNFα +/- TGFβ-induced phenotype switching

A. Confluency (left) and the number of dead cells per well (right) in GLO cells untreated or treated with TNFα treatment for 7 or 14 days with incucyte assay.

B. ZEB1 and ZEB2 mRNA expression levels in the 4 melanoma cell states from *Tsoi et al.* dataset.

C. Western blot and RT-qPCR analyses (n = 3) of ZEB1, MITF, SOX10 and SOX9 expression after 14 days of TNFα (100 ng/mL) treatment (TNF D14) in GLO cells followed by 14 days of TNFα withdrawal (TNF D14 – D14). GAPDH was used as a loading control.

D. Western blot analyses of ZEB1 and MITF expression after 14 days of TNFα (100 ng/mL) +/- TGFβ (20 ng/mL) treatment in GLO and C-09.10 cells. GAPDH was used as a loading control.

Conditions were compared **(A)** using ANOVA test (left) and Kruskal-Wallis test (right). Data are represented as Whisker plot with Tukey's method **(B)** or shown as the mean ± SEM **(C)**, P values were determined by two-tailed Mann-Whitney test **(B)** and a two-tailed paired student t test **(C)**. Differences were considered statistically significant at $*P \leq 0.05$, $**P < 0.01$ and $***P < 0.001$. ns (non-significant) means $P > 0.05$.

Supplementary Figure 2

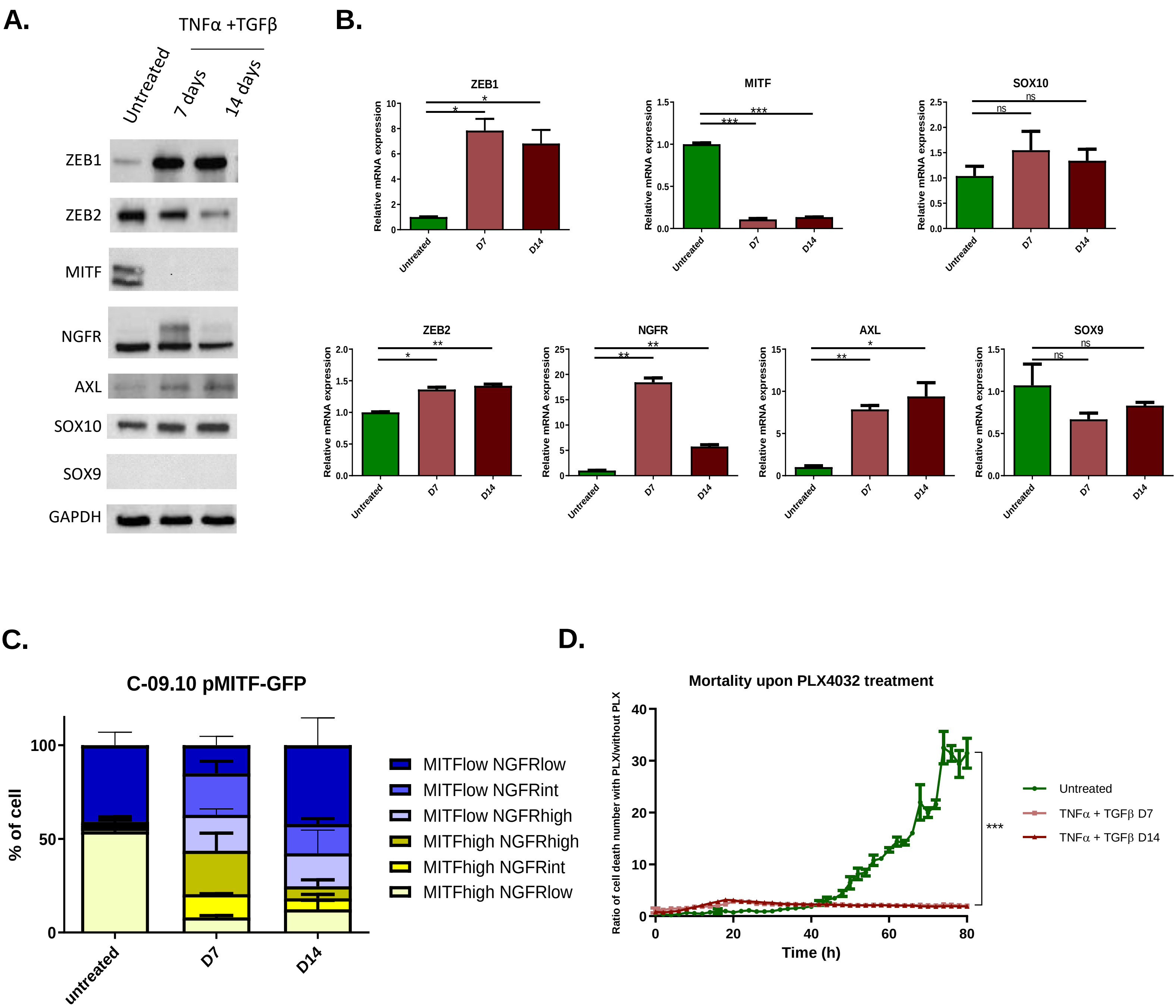

Supplementary Figure 2. Functional studies of TNFα + TGFβ-induced phenotype switching in C-09.10 cells.

Western blot **(A)** and RT-qPCR **(B)** analyses of ZEB1, ZEB2, MITF, NGFR, AXL, SOX10 and SOX9 expression after 7 and 14 days of TNFα (100 ng/mL) + TGFβ (20 ng/mL) treatment in C-09.10 cells. GAPDH was used as a loading control. Histograms represent quantitative analyses of relative expression ($n = 3$). Data are shown as the mean ± SEM. P values were determined by a two-tailed paired student t test. Differences were considered statistically significant at $^{*}P \leq 0.05$, $^{**}P < 0.01$ and $^{***}P < 0.001$. ns (non-significant) means $P > 0.05$.

C. Longitudinal intra-tumor heterogeneity characterization of MITF and NGFR expression by flow cytometry in C-09.10 pMITF-GFP cells, upon TNFα + TGFβ treatment after 7 (D7) or 14 (D14) days. NGFR was marked by anti-NGFR antibody coupled with APC. The proportion of cells with MITF high or MITF low and with NGFR high, intermediate or low status is indicated.

D. IncuCyte assay showing the relative increase in cell death upon PLX4032 (100 nM) treatment over time, in cells previously treated with TNFα + TGFβ for 7 or 14 days. P values were determined by Kruskal-Wallis test.

Supplementary Figure 3

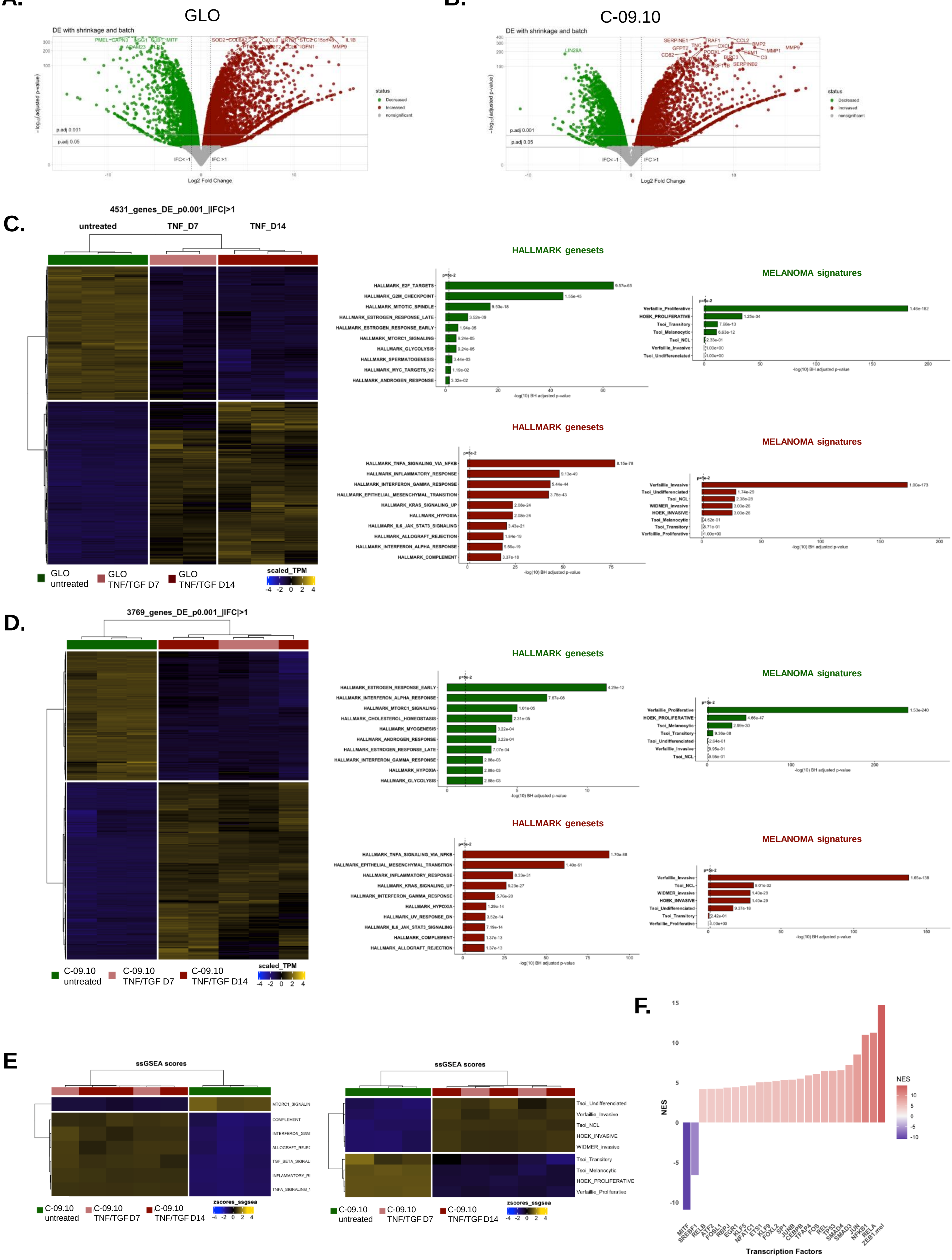

Supplementary Figure 3: RNA-seq analyses during TNFα +/- TGFb-induced phenotype switching

RNA-seq analyses of GLO and C-09.10 cells after 7 (D7) or 14 days (D14) of TNFα +/- TGFβ treatment. **A-B.** Volcano plot of differentially expressed genes in TNFα +/- TGFβ treated GLO **(A)** or C-09.10 **(B)** cells for 14 days, compared to untreated cells. Down-regulated genes are highlighted in green and up-regulated genes are highlighted in red.

C-D. Heatmap of differentially expressed genes. The most significantly enriched hallmarks and melanoma state signatures within down- and up-regulated in D14 versus untreated GLO **(C)** and C-09.10 **(D)** cells are indicated on the right.

E. Heatmap of ssGSEA scores of the most relevant hallmarks and of melanoma state signatures from Hoek, Tsoi and Verfaillie in C-09.10 cells treated with TNFα + TGFβ for 7 and 14 days.

F. Inference of transcription factors (TF) activity in gene expression data using VIPER algorithm. Barplot of DoRothEA TF Normalized Enrichment Score (NES) comparing untreated versus TNFα + TGFβ treated (D14) C-09.10 cells. The ZE1.mel regulon was added to the database.

Clustering Ward.D2 / distance: Euclidean.

Supplementary Figure 4

A.

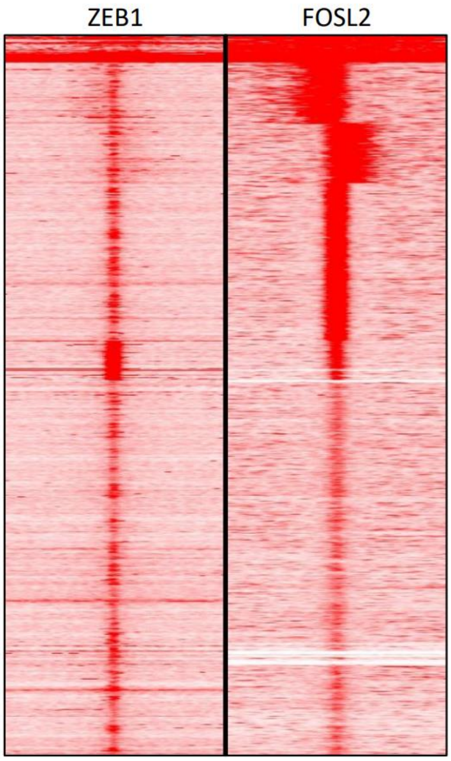

B.

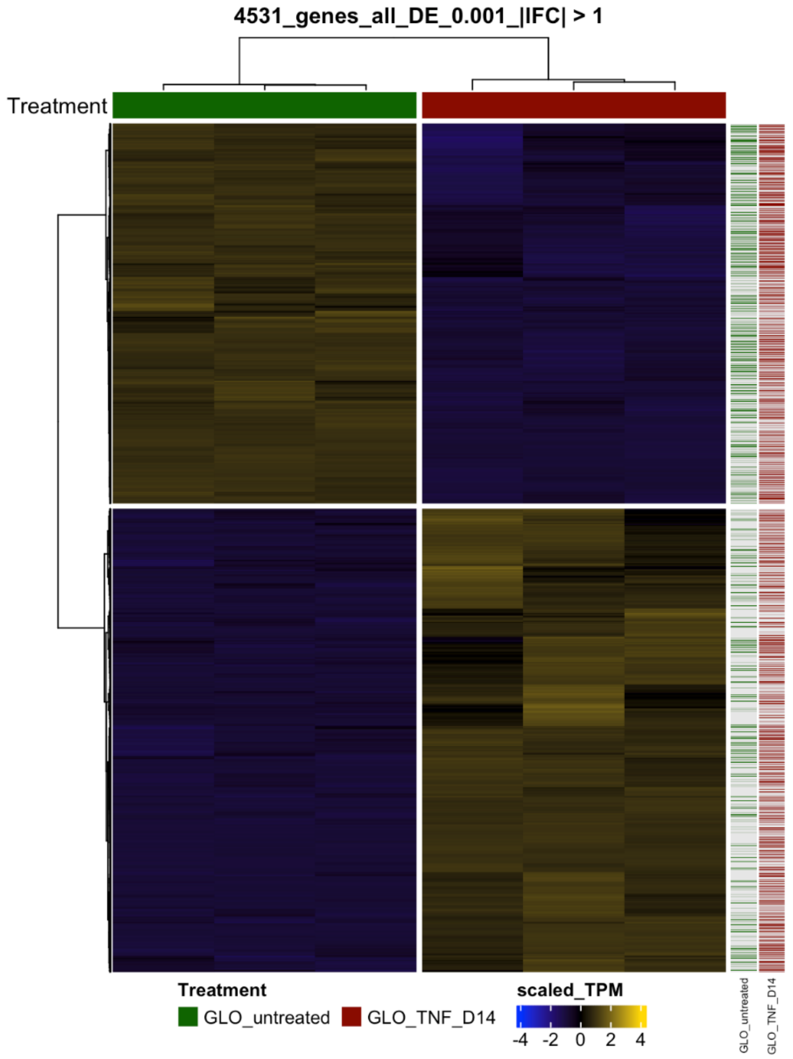

C.

	No ZEB1 peak	ZEB1 peak	P-value
All differentially expressed genes	2274 (55.2%)	1843 (44.8%)	2.19e-100
TNFα-Downregulated genes	951 (49.6%)	966 (50.4%)	2.67e-81
TNFα-Upregulated genes	1323 (60.1%)	877 (39.9%)	1.06e-23

D.

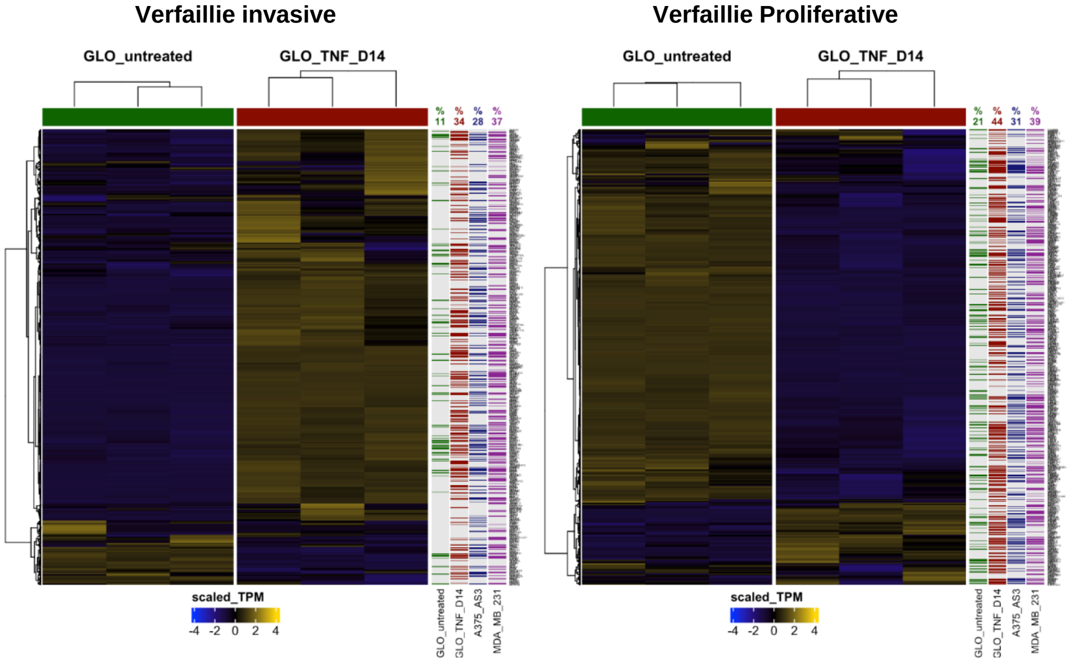

Supplementary Figure 4. Integration of ChIP-seq and RNA-seq data in GLO

A. Read density clustering of ZEB1 and FOSL2 at ZEB1-occupied loci in GLO TNF-treated (D14)(left) and in SK-MEL-147 (right).

B. Heatmap of all genes differentially expressed between untreated and TNFα-treated GLO cells at day 14. The presence of a ZEB1 peak in the gene is indicated by a green line (untreated) or a red line (TNF D14) on the right.

C. Table reprensenting the number of genes differentiatly expressed in GLO cells after TNF-α treatment. The number of associated ZEB1 peaks are indicated for all differentially expressed genes, downregulated genes and upregulated genes. The statistical enrichment of ZEB1 peaks in was tested using Fisher exact test, the p-values associated are indicated.

D. Heatmap of genes from the melanoma signatures from Verfaillie *et al.*, in untreated or TNFα-treated GLO cells at day 14. The presence of a ZEB1 peak in the gene is indicated by a green square (untreated), a red square (TNF D14), a blue square (A375-AS3) or a purple square (MDA-MB-231).

Clustering Ward.D2 / distance : Euclidean.

Supplementary Figure 5

A.

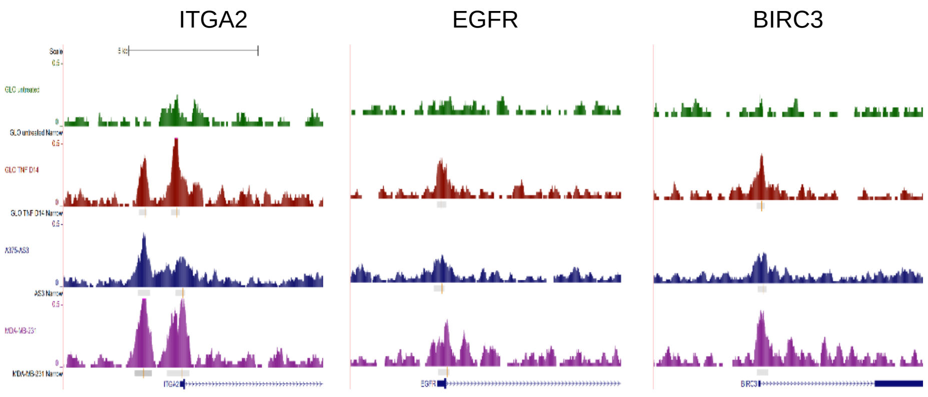

B.

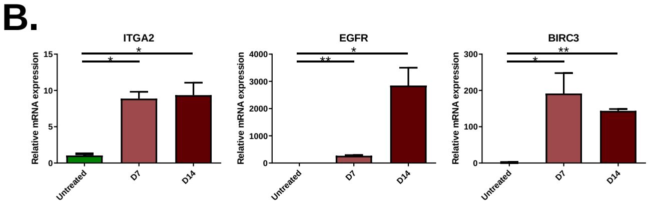

C.

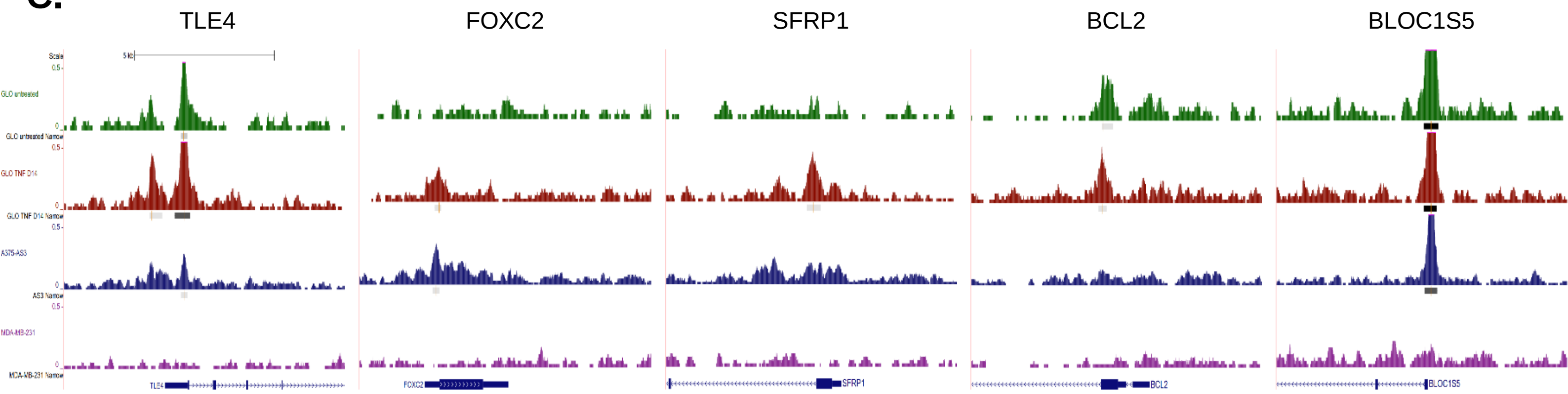

D.

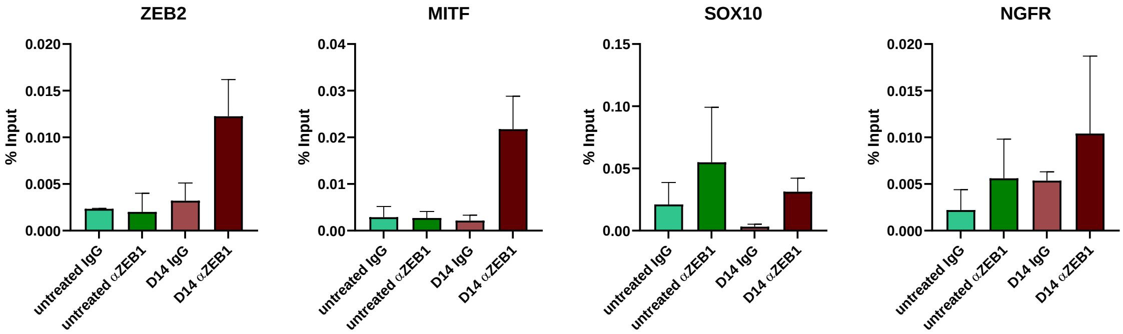

Supplementary Figure 5. ZEB1 binding on promoters of melanoma markers and direct targets related to melanoma phenotype transitions.

A. UCSC genome browser captures showing ZEB1 binding peaks in *ITGA2*, *EGFR*, *BIRC3*, promoters in untreated or TNFα-treated GLO cells at day 14, A375-AS3 cells and MDA-MB-231 cells.

B. RT-qPCR analyses showing relative expression of the corresponding genes in GLO cells upon TNFα treatment for 7 and 14 days (n = 3). Data are shown as the mean ± SEM. P values were determined by a two-tailed paired student *t* test. Differences were considered statistically significant at *P ≤ 0.05, **P < 0.01 and ***P < 0.001. ns (non-significant) means P > 0.05.

C. UCSC genome browser captures showing ZEB1 binding peaks in *TLE4*, *FOXC2*, *SFRP1, BCL2 and BLOC1S5* promoters in untreated or TNFα-treated GLO cells at day 14, A375-AS3 cells and MDA-MB-231 cells.

D. ZEB1 ChIP qPCR in C-09.10 cells treated for 14 days with TNFα + TGFβ, on the promoters of *ZEB2*, *MITF, SOX10 and NGFR.* Anti-ZEB1 (Z1) or control IgG were used for the IP. Relative promoter enrichment was normalized against chromatin inputs (n = 2).

Supplementary Figure 6

A.

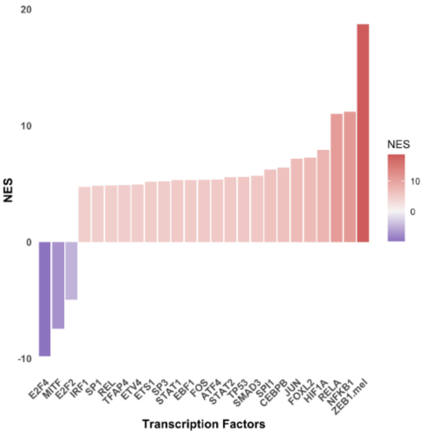

B.

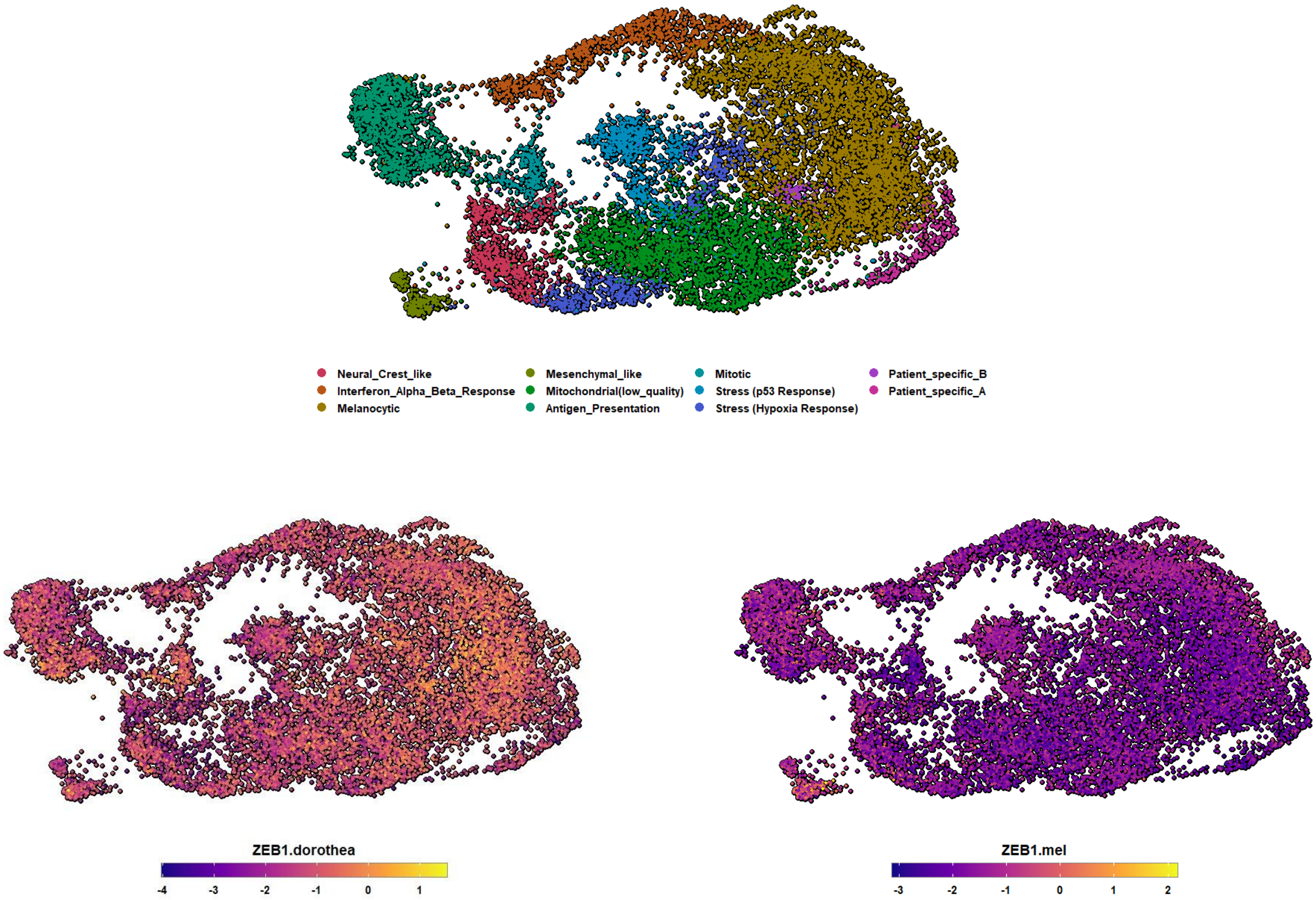

C.

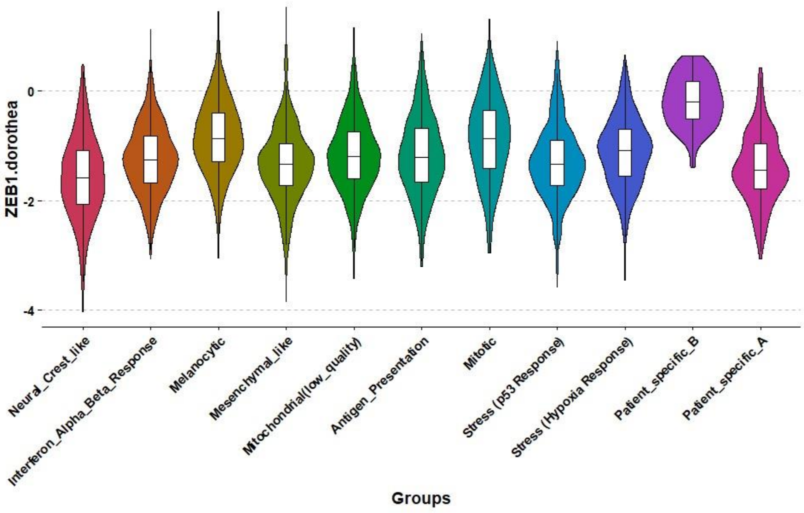

Supplementary Figure 6. ZEB1.mel regulon is more accurate than the commonly used ZEB1 pancancer regulon.

A. Inference TF activity in gene expression comparing untreated versus TNFα treated (D14) GLO cells. The ZE1.mel regulon was added to the database.

B. UMAP vizualisation of patients metastatic melanoma cells from Pozniak et al. The cell phenotype as defined in the original study as well as the transcription factor activity of the pancancer and the melanoma-specific ZEB1 regulon are indicated.

C. Violin plot of the transcription factor activity of ZEB1 pancancer regulon in the data from *Pozniak et al*.
