# Supplementary material for: ZEB1 controls a lineage-specific transcriptional program essential for melanoma cell state transitions
Source: Oncogene. 2024 Mar 22;43(20):1489–505. doi: 10.1038/s41388-024-03010-7 (PMC11090790; doi:10.1038/s41388-024-03010-7)
Supplement: Supplementary file 2 — Supplementary methods and Supp tables [file 41388_2024_3010_MOESM2_ESM.docx]

**Supplementary Material and Methods**

**RNA-seq data processing**

For careful quality controls, raw data were aligned on the human genome (GRCh38) with STAR (v2.7.8a) (Dobin et al, 2013), with the annotation of known genes from gencode v37. RNA quality control metrics were computed using RSeQC (v4.0.0) (Wang et al, 2012). Gene expression was quantified with Salmon (1.4.0) (Patro et al, 2017) on the raw sequencing reads, using the annotation of protein coding genes from gencode v37 as index.

Starting from raw counts, principal component analyses were completed with the R package *ade4* (Dray et al, 2007), selecting only the top 10% most variant genes (defined by the 10%-trimmed variance) as input data. Differential expression analyses were performed through the R package *DESeq2* (v1.34.0) (Love et al, 2014), using Wald test, sequencing batches correction and *apeglm* shrinkage estimator (v1.14.0) (Zhu et al, 2018). Heatmaps were generated with the R package *ComplexeHeatmap* (v2.10.0) (Gu Z et al, 2016). Unsupervised hierarchical clustering was performed with Euclidean distance metric and Ward.D2 clustering algorithm. Gene Set Enrichment Analyses (GSEAs) were carried out using *fgsea* R package (v1.20.0) (Korotkevich G et al, 2019) and gene lists were pre-ranked using Signal2Noise metric. Single sample GSEA (ssGSEA) scores were computed on TPM normalized data through *gsva* R package (Hänzelmann S et al, 2013). DoRothEA normalized enrichment scores were computed with the VIPER algorithm (Alvarez MJ et al, 2016) from the R package DecoupleR (Badia-i-Mompel B et al, 2022) using the log2FC of the differentially expressed genes detected by DESeq2 and on the "dorothea_hs_pancer" datatable of human TF-target interactions for cancer application (Garcia-Alonso L et al, 2019).

**ChIP-Seq data processing**

Raw sequencing data were processed with nf-core/chip-seq pipeline v.2.0.0 (https://github.com/nf-core/chipseq/tree/2.0.0). Briefly, after adapter trimming using Trimgalore, Fastq files were aligned with BWA to the human reference genome GRCh38. Reads were then filtered out in order to avoid blacklisted regions (form ENCODE), duplicates, unmapped, multiple locations, > 4 mismatches, insert size > 2kb, different chromosomes and other than FR orientation mappings. Normalized BigWig (scaled to 1 million mapped reads) were generated and peaking calling was performed with MACS2 (v2.2.7.1) (Zhang et al, 2008), independently on each IP-ZEB1 sample, considering both input and IgG immunoprecipitation as control, when available.

Genomic localization of called peaks was performed through *assignChromosomeRegion* function from ChIPpeakAnno R package (v3.28.1) (Lihua et a., 2010; Zhu et al 2013). Distance to closest TSS was defined using *annotatePeakInBatch* function with the output set as "*nearestLocation*”. Finally, peak-to-gene assignment was conducted through the *annotatePeakInBatch* function using the following options: output="overlapping", bindingRegion=c(-1000, 500), FeatureLocForDistance="TSS", select="all".

Annotation data were obtained from *TxDb.Hsapiens.UCSC.hg38.knownGene* (v3.14.0) (Team BC et al, 2019) using "transcript" as feature.

Motif enrichment analysis was conducted using findMotifsGenome function, from HOMER software (v4.11.1) (Heinz et al, 2010 <https://doi.org/10.1016/j.molcel.2010.05.004>). We selected the top first 1,000 peaks (ranked by *qvalue*) identified in GLO cells treated for 14 days with TNFα but not called in GLO untreated cells, and performed motif search (10bp) in 100-bp regions centered on each peak summit.

All analyses and statistical tests were carried out with the R software (v4.1.0) (R Core Team, 2021) and plots were generated with *ggplot2* (v3.4.3) (Wickham et al, 2016). All statistical tests were two-tailed and p-values were corrected, when indicated, with the Benjamini-Hochberg method (Benjamini et al, 1995). Enrichment of lists of genes in specific biological pathways were tested using *clusterProfiler* (v4.2.2) and *msgidbr* (v7.5.1) R packages (Yu et al, 2012; Subramanian et al. 2005). Pathway lists originated from MSigDB (Molecular Signatures Database) Hallmark (H) gene sets (Liberzon et al., 2015) together with previously published melanoma signatures. Read density clustering analysis were performed using seqMINER (Ye et al., 2011).

**References**

Dobin A, Davis CA, Schlesinger F, Drenkow J, Zaleski C, Jha S, Batut P, Chaisson M, Gingeras TR. STAR: ultrafast universal RNA-seq aligner. Bioinformatics. 2013 Jan 1;29(1):15-21. doi: 10.1093/bioinformatics/bts635. Epub 2012 Oct 25. PMID: 23104886; PMCID: PMC3530905.

Wang L, Wang S, Li W. RSeQC: quality control of RNA-seq experiments. Bioinformatics. 2012 Aug 15;28(16):2184-5. doi: 10.1093/bioinformatics/bts356. Epub 2012 Jun 27. PMID: 22743226.

Patro R, Duggal G, Love MI, Irizarry RA, Kingsford C. Salmon provides fast and bias-aware quantification of transcript expression. Nat Methods. 2017 Apr;14(4):417-419. doi: 10.1038/nmeth.4197. Epub 2017 Mar 6. PMID: 28263959; PMCID: PMC5600148.

Dray S, Dufour A (2007). “The ade4 Package: Implementing the Duality Diagram for Ecologists.” Journal of Statistical Software, 22(4), 1–20. doi: [10.18637/jss.v022.i04](https://doi.org/10.18637/jss.v022.i04).

Love MI, Huber W, Anders S (2014). “Moderated estimation of fold change and dispersion for RNA-seq data with DESeq2.” Genome Biology, 15, 550. doi: [10.1186/s13059-014-0550-8](https://doi.org/10.1186/s13059-014-0550-8).

Zhu, A., Ibrahim, J.G., Love, M.I. (2018) Heavy-tailed prior distributions for sequence count data: removing the noise and preserving large differences. Bioinformatics. [10.1093/bioinformatics/bty895](https://doi.org/10.1093/bioinformatics/bty895)

Gu Z, Eils R, Schlesner M (2016). “Complex heatmaps reveal patterns and correlations in multidimensional genomic data.” Bioinformatics.

Korotkevich G, Sukhov V, Sergushichev A (2019). “Fast gene set enrichment analysis.” bioRxiv. doi: [10.1101/060012](https://doi.org/10.1101/060012).

Hänzelmann S, Castelo R, Guinney J (2013). “GSVA: gene set variation analysis for microarray and RNA-Seq data.” BMC Bioinformatics, 14, 7. doi: [10.1186/1471-2105-14-7](https://doi.org/10.1186/1471-2105-14-7).

Zhang Y, Liu T, Meyer CA, Eeckhoute J, Johnson DS, Bernstein BE, Nusbaum C, Myers RM, Brown M, Li W, Liu XS. (2008) [Model-based Analysis of ChIP-Seq (MACS), Genome Biology, 2008;9(9):R137](http://www.ncbi.nlm.nih.gov/pubmed/18798982).

The Encode Project Consortium (2012) An integrated encyclopedia of DNA elements in the human genome. Nature 489: 57–74

Lihua J Zhu, Claude Gazin, Nathan D Lawson, Herve Pages, Simon M Lin, David S Lapointe and Michael R Green, ChIPpeakAnno: a Bioconductor package to annotate ChIP-seq and ChIP-chip data. BMC Bioinformatics. 2010, 11:237

Zhu LJ. Integrative analysis of ChIP-chip and ChIP-seq dataset. Methods Mol Biol. 2013;1067:105-24.

Team BC, Maintainer BP (2019). TxDb.Hsapiens.UCSC.hg38.knownGene: Annotation package for TxDb object(s). R package version 3.4.6.

R Core Team (2021). R: A language and environment for statistical computing. R Foundation for Statistical Computing, Vienna, Austria.

Wickham H (2016). ggplot2: Elegant Graphics for Data Analysis. Springer-Verlag New York. ISBN 978-3-319-24277-4.

Benjamini, Y., & Hochberg, Y. (1995). Controlling the False Discovery Rate: A Practical and Powerful Approach to Multiple Testing. Journal of the Royal Statistical Society. Series B (Methodological), 57(1), 289–300.

Yu G, Wang L, Han Y, He Q (2012). “clusterProfiler: an R package for comparing biological themes among gene clusters.” OMICS: A Journal of Integrative Biology, 16(5), 284-287.

Subramanian A, Tamayo P, Mootha VK, Mukherjee S, Ebert BL, Gillette MA, Paulovich A, Pomeroy SL, Golub TR, Lander ES, Mesirov JP. Gene set enrichment analysis: a knowledge-based approach for interpreting genome-wide expression profiles. Proc Natl Acad Sci U S A. 2005 Oct 25;102(43):15545-50. doi: 10.1073/pnas.0506580102. Epub 2005 Sep 30. PMID: 16199517; PMCID: PMC1239896.

Liberzon A, Birger C, Thorvaldsdóttir H, Ghandi M, Mesirov JP, Tamayo P. The Molecular Signatures Database (MSigDB) hallmark gene set collection. Cell Syst. 2015 Dec 23;1(6):417-425. doi: 10.1016/j.cels.2015.12.004. PMID: 26771021; PMCID: PMC4707969.

Ye T, Krebs AR, Choukrallah MA, Keime C, Plewniak F, Davidson I, Tora L. seqMINER: an integrated ChIP-seq data interpretation platform. Nucleic Acids Research. 2011;39:e35.

Supplementary table 1: List of antibodies for immunoblot analyses

|  | **Reference** | **Species** | **Dilution** |
| --- | --- | --- | --- |
| Anti-ZEB1 | Sigma-Aldrich, HPA027524 (RRID:AB_1844977) | Rabbit | 1/500 |
| Anti-ZEB2 | Sigma-Aldrich, HPA003456 (RRID:AB_10603840) | Rabbit | 1/500 |
| Anti-MITF | Millipore, MAB3747 (RRID:AB_570596) | Mouse | 1/500 |
| Anti-NGFR | Cell signaling, 8238S (RRID:AB_10839265) | Rabbit | 1/1000 |
| Anti-AXL | R&D Systems Bio-Techne, AF154 (RRID:AB_354852) | Goat | 1/200 |
| Anti-SOX10 | Santa cruz, sc-365692 (RRID:AB_10844002) | Mouse | 1/500 |
| Anti-SOX9 | Genetex, GTX109661 | Rabbit | 1/500 |
| Anti-GAPDH | Sigma-Aldrich, Cat# ABS16 (RRID:AB_10806772) | Rabbit | 1/20000 |

Supplementary table 2: List of primers used for RT-qPCR analyses

| Gene | Sequence | | | |
| --- | --- | --- | --- | --- |
| GAPDH | F | AGCCACATCGCTCAGACAC | R | GCCCAATACGACCAAATCC |
| ZEB1 |  | AACTGCTGGGAGGATGACAC |  | TCCTGCTTCATCTGCCTGA |
| ZEB2 |  | AAGCCAGGGACAGATCAGC |  | GCCACACTCTGTGCATTTGA |
| MITF-M |  | CATTGTTATGCTGGAAATGCTAGA |  | TGCTAAAGTGGTAGAAAGGTACTGC |
| NGFR |  | ACAAGACCTCATAGCCAGCAC |  | TGCAGCTGTTCCACCTCTTGA |
| AXL |  | GGTGGCTGTGAAGACGATGA |  | CTCAGATACTCCATGCCACT |
| SOX10 |  | GGC-TCC-CCC-ATG-TCA-GAT |  | CTG-TCT-TCG-GGG-TGG-TTG |
| SOX9 |  | GTACCCGCACTTGCACAAC |  | TCTCGCTCTCGTTCAGAAGTC |
| BIRC3 |  | TCTGGGCAGCAGGTTTACAA |  | CCCGAGATTAGACTAAGTCCCTT |
| ITGA2 |  | ACACGTGCTCTTGGTAGGTG |  | GCTGACCCAAAATGCCCTCT |
| EGFR |  | AGCAGTCACTGGGGGACTT |  | GGAGAGGAGAACTGCCAGAA |
| AQP1 |  | GGCCAGGATGAAGTCGTAGA |  | TTGCCATCGGCCTCTCTGTA |
| TNFAIP2 |  | TACCCTGACTTCAGCAAAGGC |  | CTGACGTCCAAGATGCTCCG |
| SECTM1 |  | GAGAAGCTGCATCCCAGAGGAG |  | TGTCCCAGCCTTCATTCTGAG |
| KRT7 |  | ATCAAGAACCAGCGTGCCAA |  | CACGCTCATGAGTTCCTGGT |

Supplementary table 3: List of primers used for ChIP-qPCR analyses

| Gene | Sequence | | | |
| --- | --- | --- | --- | --- |
| ZEB2 | F | AATAGAGCAGGACCTCTCCCC | R | AGGTGTCCTTTGCTATCCAGC |
| MITF |  | GGATTTCGAAGTCGGGGAGG |  | CTGCTCTTCAGCGGTTGACT |
| NGFR |  | TACGTCTCGGGGTGCAGA |  | TGCGTAGCCAGAGCTGC |
| AXL |  | CCCGGACTGTACTGTTTCTCC |  | CCGTTCCCAGACAAGGTCAG |
| SOX10 |  | AGACTCTAGGTGGGTGCGTC |  | CCCACACCAAGAGACGGTTG |

Supplementary table 4: List of antibodies for 7-color immunofluorescence multiplex analyses

| **Antibody** | **Reference** | **Dilution** | **Staining localization** |
| --- | --- | --- | --- |
| **ZEB1** | HPA02752 (RRID:AB_1844977) Sigma | 1/100 | Nuclear |
| **SOX10** | sc-365692 (RRID:AB_1084400) Santa Cruz | 1/1000 | Nuclear |
| **SOX9** | HPA001758 (RRID:AB_1080067) Sigma | 1/300 | Nuclear |
| **NGFR** | CS8238 (RRID:AB_10839265) Cell-signaling | 1/1500 | Membrane |
| **ZEB2** | HPA003456 (RRID:AB_10603840) Sigma | 1/300 | Nuclear |
| **MITF** | 284M-96 (RRID:AB_1516912) Sigma | 1/200 | Nuclear /cytoplasmic |

Supplementary table 5: Cut-off values defined for each marker of the immunofluorescence multiplex analyses

| Opal | Marker | Low | Intermediate | High |
| --- | --- | --- | --- | --- |
| 480 | SOX10 | 2 |  | 5,6 |
| 520 | ZEB1 | 3,5 | 7,2 | 11 |
| 570 | MITF | 0,435 | 0,725 | 1,45 |
| 620 | NGFR |  |  | 2 |
| 690 | SOX9 | 1,18 | 2,54 | 4,4 |
| 780 | ZEB2 | 0,3 | 0,63 | 1,1 |
